# Supplementary material for: Genetic Characteristics According to Subgroup of Acute Myeloid Leukemia with Myelodysplasia-Related Changes
Source: J Clin Med. 2022 Apr 23;11(9):2378. doi: 10.3390/jcm11092378 (PMC9105081; doi:10.3390/jcm11092378)
Supplement: Supplementary file 1 [file jcm-11-02378-s001.zip › jcm-1597592-supplementary.pdf]

Supplementary Table S1. Detected somatic variants of acute myeloid leukemia with myelodysplasia-related changes patients

Patient	Gene	Nucleotide change	Amino acid change	VAF
MRC_04	*ASXL1*	c.1900_1922del	p.E635Rfs*15	0.4256
MRC_17	*ASXL1*	c.1900_1922del	p.E635Rfs*15	0.1681
MRC_44	*ASXL1*	c.1900_1922del	p.E635Rfs*15	0.4434
MRC_02	*ASXL1*	c.1934dup	p.G646Wfs*12	0.3691
MRC_06	*ASXL1*	c.1934dup	p.G646Wfs*12	0.1931
MRC_11	*ASXL1*	c.1934dup	p.G646Wfs*12	0.3973
MRC_13	*ASXL1*	c.1934dup	p.G646Wfs*12	0.3842
MRC_28	*ASXL1*	c.1934dup	p.G646Wfs*12	0.4027
MRC_36	*ASXL1*	c.1934dup	p.G646Wfs*12	0.3982
MRC_2	*CEBPA*	c.116delT	p.L39Rfs*2	0.3503
MRC_38	*CEBPA*	c.173dup	p.H59Afs*84	0.149
MRC_01	*CEBPA*	c.268C>T	p.Q90*	0.5019
MRC_19	*CEBPA*	c.380del	p.K127Rfs*68	0.8884
MRC_12	*CEBPA*	c.605A>G	p.N202S	0.4044
MRC_11	*CSF3R*	c.1853C>T	p.T618I	0.518
MRC_03	*CSF3R*	c.2256T>G	p.Y752*	0.4513
MRC_37	*DNMT3A*	c.1481G>A	p.C494Y	0.4345
MRC_24	*DNMT3A*	c.2189G>A	p.R730H	0.524
MRC_28	*EZH2*	c.2054G>A	p.R730H	0.843
MRC_46	*FLT3*	c.1727T>C	p.L576P	0.4906
MRC_28	*FLT3*	c.1779_1793dup	p.D593_Y597dup	0.3387
MRC_09	*FLT3*	c.1806_1807ins30	p.K602_W603ins10	0.1877
MRC_08	*FLT3*	c.2503G>T	p.D835Y	0.2732
MRC_38	*FLT3*	ITD	ITD	0.03
MRC_42	*IDH1*	c.394C>G	p.R132G	0.4679
MRC_19	*IDH1*	c.394C>T	p.R132C	0.3257
MRC_24	*IDH1*	c.394C>T	p.R132C	0.3507
MRC_08	*IDH1*	c.395G>A	p.R132H	0.4791
MRC_05	*IDH2*	c.263G>A	p.R88Q	0.4422
MRC_09	*IDH2*	c.263G>A	p.R88Q	0.4862
MRC_11	*IDH2*	c.263G>A	p.R88Q	0.4617
MRC_16	*IDH2*	c.263G>A	p.R88Q	0.5085
MRC_34	*IDH2*	c.263G>A	p.R88Q	0.4818
MRC_44	*IDH2*	c.263G>A	p.R88Q	0.4848
MRC_04	*IDH2*	c.359G>A	p.R120K	0.4395
MRC_13	*IDH2*	c.359G>A	p.R120K	0.4403
MRC_20	*IDH2*	c.359G>A	p.R120K	0.4696
MRC_12	*IKZF1*	c.476A>G	p.N159S	0.4692
MRC_01	*JAK2*	c.1849G>T	p.V617F	0.6077
MRC_25	*JAK2*	c.1849G>T	p.V617F	0.3245

MRC_04	*KRAS*	c.38G>A	p.G13D	0.2091
MRC_33	*KRAS*	c.38G>A	p.G13D	0.4677
MRC_15	*MPL*	c.1544G>T	p.W515L	0.4919
MRC_33	*NPM1*	c.860_863dup	p.W288Cfs*12	0.4562
MRC_41	*NRAS*	c.35G>A	p.G12D	0.866
MRC_38	*NRAS*	c.35G>C	p.G12A	0.2691
MRC_07	*NRAS*	c.37G>T	p.G13C	0.1315
MRC_09	*PHF6*	c.821G>A	p.R274Q	0.9457
MRC_01	*PHF6*	c.941T>C	p.I314T	0.9148
MRC_08	*PTPN11*	c.179G>T	p.G60V	0.1165
MRC_02	*PTPN11*	c.214G>T	p.A72S	0.0649
MRC_28	*PTPN11*	c.214G>T	p.A72S	0.0566
MRC_38	*PTPN11*	c.214G>T	p.A72S	0.0681
MRC_12	*PTPN11*	c.226G>A	p.E76K	0.4031
MRC_34	*RUNX1*	c.1220dup	p.N407Kfs*166	0.5349
MRC_09	*RUNX1*	c.521G>A	p.R174Q	0.4918
MRC_39	*RUNX1*	c.595_596del	p.S199*	0.4308
MRC_36	*RUNX1*	c.929_930dup	p.A311Pfs*257	0.4577
MRC_09	*SRSF2*	c.281_283dup	p.A94dup	0.3579
MRC_02	*SRSF2*	c.284C>A	p.P95H	0.4579
MRC_13	*SRSF2*	c.284C>A	p.P95H	0.4386
MRC_36	*SRSF2*	c.284C>A	p.P95H	0.4802
MRC_44	*SRSF2*	c.284C>A	p.P95H	0.4442
MRC_05	*SRSF2*	c.284C>G	p.P95R	0.4313
MRC_09	*SRSF2*	c.284C>G	p.P95R	0.0769
MRC_11	*SRSF2*	c.284C>T	p.P95L	0.4913
MRC_41	*TP53*	c.273G>A	p.W91*	0.3855
MRC_41	*TP53*	c.298C>T	p.Q100*	0.3243
MRC_31	*TP53*	c.401_415del	p.F134*	0.6512
MRC_23	*TP53*	c.517G>T	p.V173L	0.5
MRC_40	*TP53*	c.524G>A	p.R175H	0.5
MRC_44	*TP53*	c.524G>A	p.R175H	0.5
MRC_29	*TP53*	c.527G>A	p.C176Y	0.4396
MRC_29	*TP53*	c.569C>T	p.P190L	0.4457
MRC_27	*TP53*	c.638G>A	p.R213Q	0.2216
MRC_42	*TP53*	c.646G>A	p.V216M	0.6057
MRC_26	*TP53*	c.706T>G	p.Y236D	0.7826
MRC_21	*TP53*	c.742C>T	p.R248W	0.7261
MRC_45	*TP53*	c.824G>A	p.C275Y	0.3866
MRC_27	*TP53*	c.832C>T	p.P278S	0.1706
MRC_03	*U2AF1*	c.101C>T	p.S34F	0.3852
MRC_19	*U2AF1*	c.470A>G	p.Q157R	0.3931

VAF: variant allele frequency, *: Termination codon

Supplementary Table S2. Comparison of gene expression according to mutation of *ASXL1*, *IDH1*/*IDH2* and *SRSF2*.

Mutation	Expression	Wild type			Mutation			*p* - value
		Median	Min	Max	Median	Min	Max	
ASXL1	*CD274*	5.43 x10^{-2}	0.49 x10^{-2}	46.4 x10^{-2}	1.48 x10^{-2}	0.97 x10^{-2}	15.3 x10^{-2}	0.020
	WT1	8.13 x10^{-2}	0.12 x10^{-2}	56.9 x10^{-2}	0.60 x10^{-2}	0.23 x10^{-2}	41.0 x10^{-2}	0.041
IDH1/2	*ABL1*	5.50 x10^{-1}	1.89 x10^{-1}	26.7 x10^{-1}	8.84 x10^{-1}	4.84 x10^{-1}	35.7 x10^{-1}	0.010
	FLT3	6.76 x10^{-1}	0.28 x10^{-1}	72.7 x10^{-1}	13.3 x10^{-1}	5.27 x10^{-1}	45.2 x10^{-1}	0.035
	RUNX1	4.69	2.09	37.2	7.49	4.06	41.9	0.007
	IRF8	1.83	0.12	34.9	0.66	0.15	3.67	0.014
	MECOM	8.79 x10^{-3}	0.75 x10^{-3}	1.27	3.80 x10^{-3}	0.27 x10^{-3}	99.2 x10^{-3}	0.017
	MYH11	7.49 x10^{-3}	1.97 x10^{-3}	77.9 x10^{-3}	3.52 x10^{-3}	0.72 x10^{-3}	44.3 x10^{-3}	0.033
SRSF2	*CD274*	5.43 x10^{-2}	0.49 x10^{-2}	46.4 x10^{-2}	1.60 x10^{-2}	0.97 x10^{-2}	3.79 x10^{-2}	0.026
	IRF8	1.56	0.15	34.9	0.59	0.12	2.51	0.024
	MECOM	8.16 x10^{-3}	0.36 x10^{-3}	1.27	2.89 x10^{-3}	0.27 x10^{-3}	9.34 x10^{-3}	0.049
	PDCD1	2.10 x10^{-2}	0.29 x10^{-2}	22.6 x10^{-2}	0.76 x10^{-2}	0.29 x10^{-2}	4.14 x10^{-2}	0.045

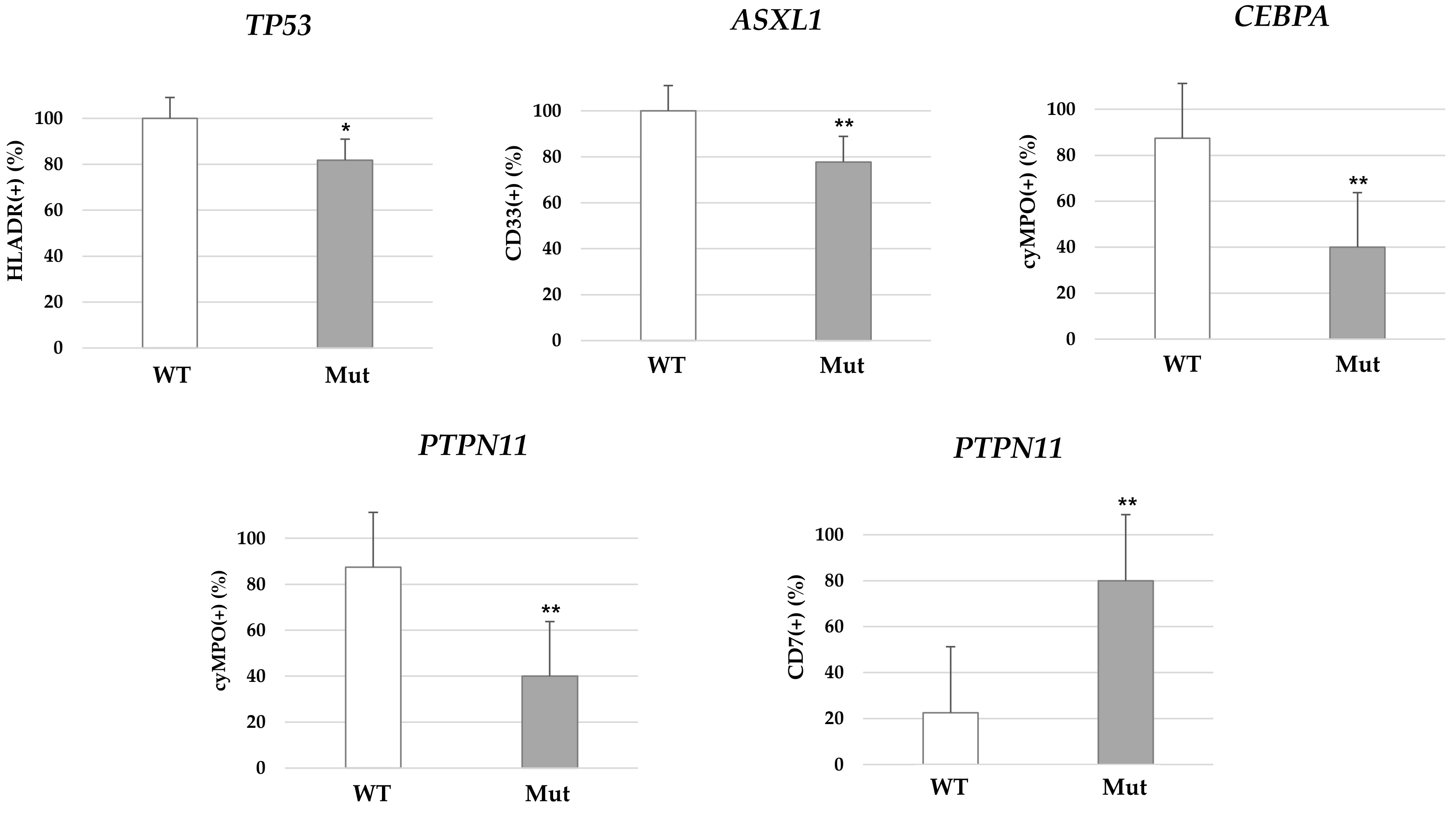

Supplementary Figure S1. Comparison of immunophenotype according to gene mutations. WT: wild type, Mut: mutation, *: *p*-Value < 0.05, **: *p*-Value < 0.01

Supplementary Table S3. Summary of genetic mutations in studies including acute myeloid leukemia with myelodysplasia-related changes.

Ref.	No. patients	Diagnosis	Age	Study period	Sequencing methods	
1	48/37	AML-MRC/No-AML-MRC	72/63	NP	Sanger sequencing	*ASXL1* (35%), *RUNX1* (17%), *TET2* (15%), *IDH1/2* (25%), *DNMT3A* (8%), *NPM1* (8%), *FLT3* (2%): High frequency of *ASXL1* mutations and a low rate of *NPM1*, *FLT3*, and *DNMT3A* mutations
2	125	AML-MRC	71	NP	Sanger sequencing	*ASXL1* (21%), *RUNX1* (12%), *DNMT3A* (9%), *NPM1* (3%), *FLT3* (7%), *TP53* (22%): *ASXL1* and *TP53* mutations identify two molecular subgroups among AML-MRCs, with specific poor prognosis
3	36/57	AML-MRC/Other AML	55	NP	Sanger sequencing - *TP53*<br>Targeted sequencing	*NRAS* (12.3%), *RUNX1* (10.8%), *U2AF1* (9.2%), *DNMT3A* (9.2%), *SF3B1* (7.7%), *SRSF2* (7,7%), *TP53* (7.7%), *ASXL1* (6.2%), *TET2* (6.2%), *FLT3*-ITD (6.2%)
4	19/149	AML-MRC/Other de novo AML	NP/60.6	2009-2016	Targeted sequencing	Cohesin pathway mutation (specifically *STAG2*) associated with megakaryocytic dysplasia (11%)
5	61/107	AML-MRC/AML-NOS	61/58	2008-2019	Sanger sequencing - *ASXL1*<br>Targeted sequencing	*ASXL1* (31%/4.3%): specific characteristics including morphological dysplasia
6	415	AML-MRC	70	2013-2018	Targeted sequencing	*TP53* (39%), *ASXL1* (20%), *NRAS* (17%), *DNMT3A* (16%), *SRSF2* (14%), *TET2* (14%), *U2AF1* (14%)
7	179	AML-MRC	67.4	2005-2018	Multiplex RT-PCR - *NPM1*, *FLT3*-ITD<br>Targeted sequencing	AML-MRC-M (n=9): *ASXL1* (56%), *DNMT3A* (44%), *U2AF1* (33%), *GATA2* (33%), *SF3B1* (33%).
8	99/70<br>/167/310	pAML-MRC/sAML-MRC<br>/AML wo MRC/MDS	67/69<br>/68/72	2013-2015	Targeted sequencing	pAML-MRC: *TP53* (28%), *DNMT3A* (19%), *ASXL1* (17%), *RUNX1* (8%), *SRSF2* (10%)<br>sAML-MRC: *ASXL1* (30%), *RUNX1* (28%), *SRSF2* (21%), *TP53* (20%), *DNMT3A* (15%)
9	72/105	AML-MRC/Non-AML-MRC	68/62	2017-2020	Targeted sequencing	*TP53* (45.8%), *ASXL1* (18.1%), *SRSF2* (15.3%), *RUNX1* (9.7%), *U2AF1* (8.3%), *TET2* (8.3%), *DNMT3A* (8.3%)

Ref.: reference, No. patients: the number of patients, AML-MRC: acute myeloid leukemia with myelodysplasia-related changes, NOS: not otherwise specified, pAML-MRC: primary AML-MRC, sAML-MRC: secondary AML-MRC, AML wo MRC/MDS: AML without MRC/MDS, MDS: myelodysplastic syndrome, NP: not presented.

References of Supplementary Table S3

1. Devillier, R., et al. (2012). Acute myeloid leukemia with myelodysplasia-related changes are characterized by a specific molecular pattern with high frequency of ASXL1 mutations. Am J Hematol 87(7): 659-662.
2. Devillier, R., et al. (2015). Role of ASXL1 and TP53 mutations in the molecular classification and prognosis of acute myeloid leukemias with myelodysplasia-related changes. Oncotarget 6(10): 8388-8396.
3. Ohgami, R. S., et al. (2015). Next-generation sequencing of acute myeloid leukemia identifies the significance of TP53, U2AF1, ASXL1, and TET2 mutations. Mod Pathol 28(5): 706-714.
4. Weinberg, O. K., et al. (2018). Association of mutations with morphological dysplasia in de novo acute myeloid leukemia without 2016 WHO Classification-defined cytogenetic abnormalities. Haematologica 103(4): 626-633.
5. Prats-Martín, C., et al. (2020). ASXL1 mutation as a surrogate marker in acute myeloid leukemia with myelodysplasia-related changes and normal karyotype. Cancer Med 9(11): 3637-3646.
6. Montalban-Bravo, G., et al. (2020). Outcomes of acute myeloid leukemia with myelodysplasia related changes depend on diagnostic criteria and therapy. Am J Hematol 95(6): 612-622.
7. Jiang, G., et al. (2020). Acute myeloid leukemia with myelodysplasia-related changes diagnosed with multilineage dysplasia alone demonstrates a superior clinical outcome. Hum Pathol 104: 117-126.
8. Badar, T., et al. (2020). Interrogation of molecular profiles can help in differentiating between MDS and AML with MDS-related changes. Leuk Lymphoma 61(6): 1418-1427.
9. Yu, J., et al. (2021). Mutational profiling of myeloid neoplasms associated genes may aid the diagnosis of acute myeloid leukemia with myelodysplasia-related changes. Leuk Res 110: 106701.
